# Linking Gut Microbiota and Stereotypic Behavior of Endangered Species Under Ex Situ Conservation: First Evidence from Sun Bears

**DOI:** 10.3390/ani15030435

**Published:** 2025-02-04

**Authors:** Xiaobing Chen, Wenqi Chen, Xinyu Guo, Sheng Zhang, Bo Xu, Hong Wu, Dapeng Zhao

**Affiliations:** 1 College of Life Sciences, Tianjin Normal University, Tianjin 300387, China; 2 Tianjin Zoo, Tianjin 300381, China

**Keywords:** carnivore, intestinal flora, animal welfare, ex situ conservation, behavioral correlation

## Abstract

Integrative conservation research on animal behavior and nutritional health can contribute to the ex situ conservation of endangered species. Stereotypic behavior, a repetitive behavior without practical function, is associated with animal welfare in its manner and frequency for captive animals. Exploring the potential relationship between stereotypic behavior and internal factors, such as intestinal flora, could improve ex situ conservation, especially for endangered species. In this study, we analyzed the typical behavior characteristics of the endangered sun bears (*Helarctos malayanus*) under captive conditions based on the behavior sampling method. The seasonal and annual changes in the intestinal flora of *H. malayanus* in captivity were studied by 16S rDNA high-throughput sequencing technology based on non-invasive fecal sample collection. This study provides the first evidence of a potential association between the gut microbiota and stereotypic behavior characteristics of captive *H. malayanus*. The results can significantly improve our understanding of the stereotypical behavior of *H. malayanus* in captivity and contribute to the captive breeding and conservation efforts of this endangered species.

## Simple Summary

Stereotypic behavior could provide valuable insights into the stress and welfare of animals in captivity. This study focuses on sun bears as the focal subjects and is the first to correlate the structural characteristics of intestinal flora with stereotyped behavior for endangered species under ex situ conservation conditions. Significant correlations were found between the occurrence frequency of pacing behavior, one typical type of stereotypical behavior, and the abundance of certain gut microbiota at both the phylum and genus levels. It suggests the intestinal flora may interact with stress-related behaviors in captive sun bears, which has important implications for the scientific conservation of endangered species.

## 1. Introduction

Stereotypic behavior is known as a repetitive, unchanging pattern of behavior with no apparent goal or function [1,2]. Stereotypic behavior exists in human beings [3] as well as other animals [4,5], and can have adverse psychological and physiological effects [6]. ASD is a heterogeneous neurodevelopmental disorder, including social functioning, stereotyped patterns of behaviors, narrowed interests, and elevated anxiety [7]. Stereotypic behavior is the main core symptom of autism spectrum disorder (ASD) [8,9].

Stereotypic behavior also appears in non-human animals living in captivity [10,11,12,13,14]. In comparison to the self-sufficiency required in the wild, captive animals live in a relatively simple environment with both the abundance of food resources and the absence of potential predators; thus, they do not need to expend the extra energy foraging for food and avoiding predators like their wild counterparts [13]. The captive environment is often related to sources of stress (e.g., characteristics of living environment, food access), with reduced opportunities for natural behavioral expression [4]. For example, Quirke et al. (2012) found that captive cheetahs (*Acinonyx jubatus*) living in small spaces often display more stereotypic behaviors (pacing and repeatedly licking fences) [15]. Marinath et al. (2019) found that feeding the whole live prey could reduce the stereotypic behavior of captive jungle cats (*Felis chaus*) [16]. The presence of stereotypic behavior reduces the productivity and value of captive animals [17], while also impairing their reproductive capacity to a certain degree [1]. Nowadays, stereotypic behavior is commonly applied as one behavioral indicator to assess chronic or acute stress and health in captive individuals, which could provide valuable insights into the animal welfare in captivity [18]. Therefore, it is particularly crucial to further explore the expression and potential mechanism of stereotypic behavior in captive animals, especially endangered species [13,19].

Increasing data have shown that interspecific and interindividual variability in stereotypic behavior exists among captive animals [20,21,22,23,24]. For instance, in a stereotypic behavior survey including more than thirty carnivore species, bears had the highest incidence and prevalence in congeners compared to other families (e.g., canids, felines), especially in captivity, where bears are relatively prone to stereotypic behavior [14]. Current studies have documented many kinds of stereotypic behaviors in bears (e.g., pacing, head rocking, stereotypical swimming, tongue flapping), among which pacing behavior is the most prominent manifestation [22]. Therefore, this study primarily focuses on the correlation analysis between pacing behavior and the gut microbiota, using this as an example to uncover the relationship between stereotypic behavior and animal health.

Besides the influence of external factors, current evidence has shown that behavior is known to be associated with the microbiome. For example, the microbiome–gut–brain axis provides a direct link between mental health and the microbiome [25]. The microbiome–gut–brain axis has been identified as a potential way to influence ASD symptoms and has been associated with various abnormal behaviors (e.g., stereotypic behavior, social withdrawal) [26]. It has been shown that the composition of intestinal flora can affect the expression of stereotypic behavior. Sharon et al. (2017) found that the sterile mice transplanted with the intestinal flora of children with ASD showed increased repetitive behaviors and decreased communication [27]. Niu et al. (2019) found that the relative abundance of the genera *Bacteroidetes*, *Bacteroides*, *Bifidobacterium*, and *Ruminococcus* in the ASD group was significantly lower than that in the normal group, indicating that specific bacteria groups may affect the expression of ASD [28]. In addition, Pan et al. (2021) found that the intestinal flora of the abnormal group of the rhesus macaque (*Macaca mulatta*) with stereotype behavior was significantly different from that of the normal group [18]. Therefore, investigating the potential correlation between animal stereotypic behavior and their gut microbiota has important implications for understanding species-specific stereotypes and implementing effective interventions. To date, comparative studies on the gut microbiota of bears are relatively limited and mainly focus on the basic composition of the gut microbiota, seasonal changes, geographical differences, and phylogeny [29,30,31]. At present, there is no study related to the association between stereotypic behavior and intestinal flora of bears.

In this study, based on the scanning sampling method, non-invasive sampling technology, and 16S rDNA high-throughput sequencing technology from fecal samples, we investigated both seasonal and annual variations in the stereotypic behavior of captive sun bear (*Helarctos malayanus*) living in Tianjin Zoo. Meanwhile, our study presents the first evidence of the potential association between stereotypic behavior and intestinal flora for this endangered species in captivity. The findings in the study could significantly enhance our comprehension of stereotypic behavior characteristics in sun bears while contributing to artificial breeding efforts and conservation initiatives for these endangered species.

## 2. Materials and Methods

### 2.1. Study Site and Subjects

This study focused on sun bears living in Tianjin Zoo, which is located in North China, with four distinct seasons: spring (from March to May), summer (from June to August), autumn (from September to November), and winter (from December to February).

The sun bears selected in this study included six females and two males (all animals were healthy), living in the breeding center of Tianjin Zoo (Table 1). According to the longevity and sexual maturity age of sun bears, the age of <12 years is defined as adult and >12 years as old age [32]. These areas are not open to visitors and each individual was reared separately. Natural light and free water were provided, and the daily diet mainly included carrots, steamed buns, chicken racks, bread, eggs, and seasonal fruit twice a day (Table S1).

### 2.2. Behavioral Observation and Data Analysis

One classic method of behavioral observation of captive bears is scan sampling [6,13]. Scan sampling describes which behavior an animal exhibits at a fixed time interval. Our study applied the scanning sampling method and observational data were recorded with 5 min intervals. The recording period of each observation day was mainly between 10:00 and 15:00. The four behavioral observation periods consisted of the autumn of 2020 and the spring, summer, and autumn of 2023. Observation days and frequencies corresponding to the observation hours of each individual during each observation period are shown in Table 1. All observations were performed by the same people, positioned in the same area [33].

Based on the early behavioral observations for focal subjects as well as the behavioral research findings of sun bears and other bears [13,34,35], the behaviors of sun bears in this study were divided into three categories: resting behavior, active behavior, and stereotypic behavior [36,37] (Table 2). All data were collated and summarized in Excel 2019, and the frequency of each behavior was calculated as a proportion of observations (the number of scans per behavior/total number of scans). Friedman rank-sum tests and Wilcoxon signed-rank tests were used to investigate seasonal differences in the occurrence frequency of one certain type of behavior. Wilcoxon signed-rank test was used to explore inter-year differences in the occurrence frequency of one certain type of behavior.

### 2.3. Fecal Sample Collection and Experimental Analysis

Based on non-invasive sampling technology, fresh fecal samples were collected and put into sterile 5 mL EP tubes. Then, they were brought back to the laboratory for storage at −80 °C until analysis. Six fecal samples were collected from six individuals, except DL and TT, on 24 September 2020, and numbered GG-1-F-Y, LL-1-F-Y, BB-1-M-Y, ZZ-1-M-O, SJZ-1-F-O, and HH-1-F-O (F: female, M: male; Y: young, and O: old). A total of eight fecal samples were collected from all individuals on 25 November 2023, and numbered TT-2-F-Y, GG-2-F-Y, LL-2-F-Y, BB-2-M-Y, ZZ-2-M-O, SJZ-2-F-O, DL-2-F-O, and HH-2-F-O (Table S2).

The total genomic DNA was extracted by the TIANamp Stool DNA Kit (Tiangen Biochemical Technology, Beijing, China). The concentration and quality of DNA samples were determined by the Nano Drop 2000 spectrophotometer (Thermo Fisher Scientific, Waltham, MA, USA). After detection by 1% agarose gel electrophoresis, the fecal samples were selected for the high-throughput sequencing of the V3~V4 region of the 16S rDNA gene. The forward primer 338F (5′-ACTCCTACGGGAGGCAGCA-3′) and the reverse primer 806R (5′-GGACTACHVGGGTWTCTAAT-3′) were used for PCR amplification. The purified PCR amplification products were sequenced by Shanghai Majorbio Bio-pharm Technology Co., Ltd. (Shanghai, China) on the Illumina MiSeq platform PE 300, PE250.

The sequence was determined through the high-throughput sequencing platform, and the diversity and community structure differences of the microbial community were studied by bioinformatics analysis software, and the species abundance, classification, and phylogenetic development were determined using RDP classifier (version 2.11). The Bayesian algorithm was used to analyze the sequences representing operational taxonomic units (OTUs) at the 97% similarity level. Qiime (version 1.9.1) was used to generate the abundance table of each taxonomic level. Both Wilcoxon rank-sum tests and Wilcoxon signed-rank tests were applied to investigate the inter-group difference in the abundance of gut microbiota at the phylum and genus levels, respectively. Based on the Mothur (version 1.30.2) virtual software, Welch’s *t* tests were used to compare the alpha diversity indices, including the Ace, Chao, Shannon, and Simpson indices. Principal coordinates analyses (PCoAs) based on unweighted UniFrac and weighted UniFrac analysis were used to explore inter-group differences in community structure by means of the R language virtual software (version 3.3.1).

### 2.4. Correlation Between Stereotypic Behavior and Gut Microbiota

Spearman’s rank correlation analysis was used to explore the potential relationship between the occurrence frequency of stereotypic behavior and the abundance of one certain phylum/genus. The top ten phyla and top thirty genera were chosen for the correlation analysis (all of the gut microbiota were included). Correlation plots and fitting curves were constructed via the Origin 2023 software.

All data statistical analyses were conducted using SPSS 27, Excel 19, and the Major Bioscience Cloud Analysis Platform (https://cloud.majorbio.com) of Shanghai Majorbio Bio-pharm Technology Co., Ltd. The significance level and high significance level were defined as *p* ≤ 0.05 and *p* ≤ 0.01, respectively.

## 3. Results

### 3.1. Variation in Behavioral Categories

In this study, we focused on recording three behavioral categories, including stereotypic behavior, active behavior, and resting behaviors.

**Figure 1:**
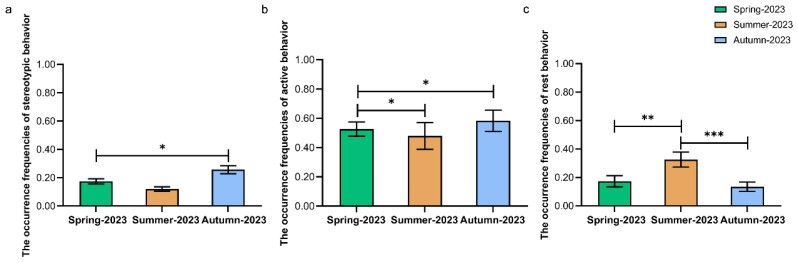
Seasonal comparison in 2023 regarding the occurrence frequencies of three categories of behaviors. Seasonal comparison in 2023 regarding the occurrence frequencies of stereotypic behavior (**a**), active behavior (**b**), and rest behavior (**c**). The horizontal axis represents different seasons in 2023. The vertical axis indicates the frequency of each behavior, which was calculated as a proportion of observations (the number of scans per behavior/total number of scans). * *p* ≤ 0.05, ** *p* ≤ 0.01, *** *p* ≤ 0.001. N = 6 per group.

For the observation in 2023 (N = 6: LL, BB, ZZ, SJZ, DL, and HH), there were significant seasonal differences in the occurrence frequency of stereotypic behavior in sun bears (Friedman rank-sum test: *χ*^2^ = 6.500, *p* = 0.039). The occurrence frequency of stereotypic behaviors in autumn was significantly higher than that in spring (Wilcoxon signed-rank test: *Z* = −2.023, *p* = 0.043). The occurrence frequency of stereotypic behaviors in summer was lower than that in spring (Wilcoxon signed-rank test: *Z* = −1.461, *p* = 0.144) and in autumn (Wilcoxon signed-rank test: *Z* = −1.826, *p* = 0.068), without reaching the significance level (Figure 1a).

We found no significant seasonal difference in the occurrence frequency of active behavior of sun bears in 2023 (Friedman rank-sum test: *χ*^2^ = 4.000, *p* = 0.135) (Figure 1b).

In 2023, significant seasonal differences were found in the occurrence frequency of resting behavior of sun bears (Friedman rank-sum test: *χ*^2^ = 16.083, *p* < 0.001). The occurrence frequency of resting behavior in summer was extremely significantly higher than that in spring (Wilcoxon signed-rank test: *Z* = −3.143, *p* = 0.002) and that in autumn (Wilcoxon signed-rank test: *Z* = −3.286, *p* = 0.001) (Figure 1c).

**Figure 2:**
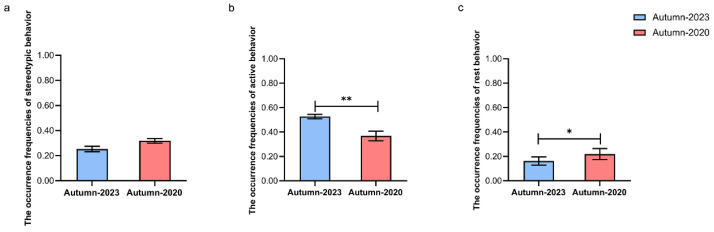
Inter-year comparison in the same season on occurrence frequencies of three categories of behaviors. Inter-year comparison in autumn on occurrence frequencies of stereotypic behavior (**a**), active behavior (**b**), and rest behavior (**c**). The horizontal axis represents different years in autumn. The vertical axis indicates the frequency of each behavior, which was calculated as a proportion of observations (the number of scans per behavior/total number of scans). * *p* ≤ 0.05, ** *p* ≤ 0.01. N = 5 per group.

Compared to 2020 and 2023 (N = 5: LL, BB, ZZ, SJZ, and HH), we found that: (1) the occurrence frequency of active behavior in the autumn of 2023 was significantly higher than that of same behavior in the autumn of 2020 (Wilcoxon signed-rank test: *Z* = −2.866, *p* = 0.004) (Figure 2b); (2) the occurrence frequency of resting behavior in the autumn of 2020 was significantly higher than that of same behavior in the fall of 2023 (Wilcoxon signed-rank test: *Z =* −2.489, *p* = 0.013) (Figure 2c).

### 3.2. Composition of and Variation in Gut Microbiota of Sun Bears

After high-throughput sequencing, 1,120,217 optimized sequences with an average sequence length of 440 bp were obtained after the removal of low-quality reads. According to the OTU clustering with 97% similarity, a total of 17 phyla, 28 classes, 64 orders, 116 families, 199 genera, and 264 species were obtained. Among them, 15 phyla, 22 classes, 48 orders, 82 families, 123 genera, and 151 species were obtained from the 2020 sample group. Meanwhile, a total of 15 phyla, 23 classes, 54 orders, 91 families, 156 genera, and 201 species were obtained from the 2023 sample group. The commonality of two sample groups included 13 phyla, 17 classes, 38 orders, 57 families, 80 genera, and 98 species. Compared to the two sample groups, there were 13 shared phyla, and both the 2023 sample group and 2020 sample group had two unique bacteria; there were 80 shared genera levels, and the 2023 sample group had 33 more unique genera than in 2020; and there was 116 shared OTU levels, and the 2023 sample group had 146 more unique OTUs than in 2020 (Figure S1).

#### 3.2.1. The Structural Composition of Gut Microbiota at the Phylum Level

**Figure 3:**
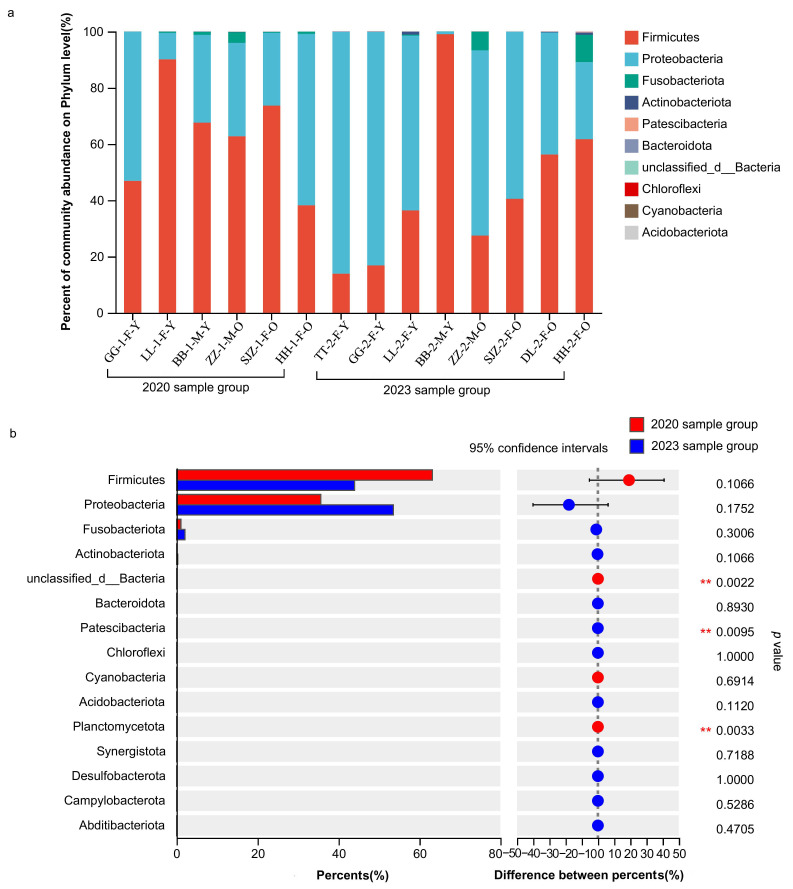
The gut microflora composition and comparison of the relative abundance at the phylum level. (**a**) The gut microflora composition at the phylum level. The gut microflora with different phyla in different colors correspond to the legend on the right; the horizontal axis represents different sample groups, and the vertical axis represents the percentage of community abundance on the phylum level. (F: female, M: male; Y: young, and O: old). (**b**) The gut microflora comparison of relative abundance at the phylum-level. The vertical axis represents different gut microflora at the phylum level, the horizontal axis represents the percentage of different phyla and the difference between these percentages for the 2020 sample group and 2023 sample group, and the *p* value (Wilcoxon rank-sum tests) corresponds to the legend on the right. ** *p* ≤ 0.01.

The dominant phyla of the 2020 sample group included Firmicutes (63.39%), Proteobacteria (35.33%), and Fusobacteriota (1.06%), while the dominant phyla of the 2023 sample group included Firmicutes (53.58%), Proteobacteria (43.95%), and Fusobacteriota (2.08%) (Figure 3a). The common dominant phyla of the two sample groups were Firmicutes, Proteobacteria, and Fusobacteriota (Table S3).

Based on the Wilcoxon rank-sum test, we found that: (1) the abundances of Planctomycetota (*p* = 0.0033) and unclassified_Bacteria (*p* = 0.0022) in the 2023 sample group were significantly lower than those in the 2020 sample group; (2) the abundance of Patescibacteria (*p* = 0.0095) in the 2023 sample group was significantly higher than that in the 2020 sample group (Figure 3b). For the common individuals from two sample groups (N = 6: GG, LL, BB, ZZ, SJZ, and HH), we found that there was no inter-year significant difference in the abundance of all common dominant phyla of two sample groups (Firmicutes: *Z* = −1.363, *p* = 0.173; Proteobacteria: *Z* = −0.943, *p* = 0.345; Fusobacteriota: *Z* = −0.405, *p* = 0.686).

Compared to the old and young groups in 2020 and 2023, based on the Wilcoxon signed-rank test, we found that there was no significant difference in phyla (all *p* > 0.05).

#### 3.2.2. The Structural Composition of Gut Microbiota at the Genus Level

**Figure 4:**
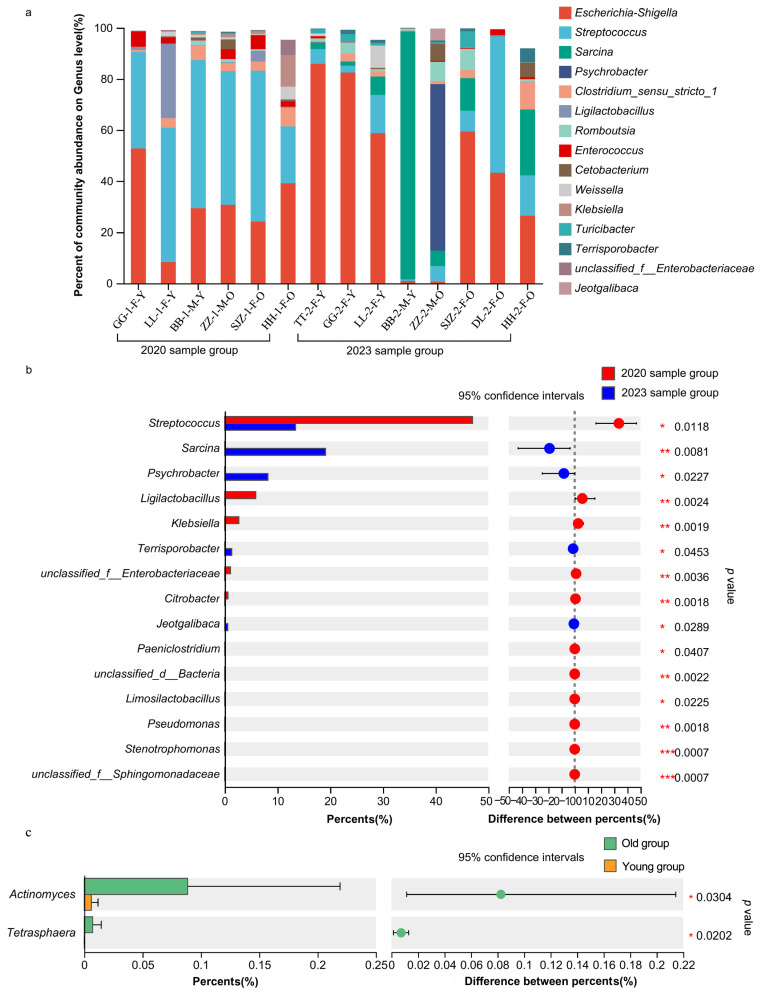
The gut microflora composition and comparison of the relative abundance at the genus level. (**a**) The gut microflora composition at the genus level. The gut microflora with different genera in different colors correspond to the legend on the right; the horizontal axis represents different sample groups, and the vertical axis represents the percent of community abundance at the phylum level (F: female, M: male; Y: young, and O: old). (**b**) The gut microflora comparison of the relative abundance at the genus level between the 2020 sample group and the 2023 sample group. (**c**) The gut microflora comparison of the relative abundance at the genus level between the old group and young group. The vertical axis represents different gut microflora at the genus level, the horizontal axis represents the percentages of different genera and the difference between these percentages for different groups, and the *p* value (Wilcoxon rank-sum tests) corresponds to the legend on the right. * *p* ≤ 0.05, ** *p* ≤ 0.01, *** *p* ≤ 0.001.

The dominant genera of the 2020 sample group included *Streptococcus* (46.84%), *Escherichia-Shigella* (30.65%), *Ligilactobacillus* (5.91%), *Clostridium_sensu_stricto_1* (3.93%), *Enterococcus* (3.43%), *Klebsiella* (3.25%), *Weissella* (1.56%), and *Cetobacterium* (1.06%). The dominant genera of the 2023 sample group included *Escherichia-Shigella* (44.73%), *Sarcina* (19.09%), *Streptococcus* (13.44%), *Psychrobacter* (8.20%), *Romboutsia* (3.01%), *Clostridium_sensu_stricto_1* (2.55%), *Turicibacter* (1.60%), *Cetobacterium* (1.57%), *Terrisporobacter* (1.34%), and *Weissella* (1.25%) (Figure 4a). The common dominant genera included *Escherichia-Shigella*, *Streptococcus*, *Clostridium_sensu_stricto_1*, *Weissella*, and *Cetobacterium*. The unique dominant genera of the 2020 sample group were *Ligilactobacillus* (5.91%), *Enterococcus* (3.43%), and *Klebsiella* (3.25%), while the unique dominant genera of the 2023 sample group were *Sarcina* (19.09%), *Psychrobacter* (8.20%), *Romboutsia* (3.01%), *Turicibacter* (1.60%), and *Terrisporobacter* (1.34%) (Table S3).

Based on the Wilcoxon rank-sum tests, for the total individuals from the two sample groups, it was found that: (1) the abundance of *Stenotrophomonas* (*p* = 0.0007), *unclassified_f__Sphingomonadaceae* (*p* = 0.0007), *unclassified_f__Enterobacteriaceae* (*p* = 0.0036), *Ligilactobacillus* (*p* = 0.0024), *Klebsiella* (*p* = 0.0019), *Citrobacter* (*p* = 0.0018), *unclassified_d__Bacteria* (*p* = 0.0022), and *Pseudomonas* (*p* = 0.0018) in the 2023 sample group were significantly lower than those in the 2020 sample group; (2) the abundance of *Sarcina* (*p* = 0.0081) in the 2023 sample group was significantly higher than that in the 2020 sample group (Figure 4b).

In terms of the common dominant genera, based on the Wilcoxon signed-rank tests, for the common individuals from two sample groups (N = 6: GG, LL, BB, ZZ, SJZ, and HH), this study found that: (1) the abundance of *Streptococcus* in 2023 (Z = −2.201, *p* = 0.028) was significantly lower than that in 2020; (2) there was no significant inter-year difference on the abundances of *Escherichia-Shigella* (Z = −0.734, *p* = 0.463), *Clostridium_sensu_stricto_1* (Z = −0.314, *p* = 0.753), *Cetobacterium* (Z = −0.405, *p* = 0.686), and *Weissella* (Z = −0.943, *p* = 0.345).

Compared to the old and young groups in 2020 and 2023, based on the Wilcoxon signed-rank test, we found that: (1) there was no significant difference in genera between the old and young groups in 2020 (all *p* > 0.05); (2) the abundances of *Actinomyces* (*p* = 0.0304) and *Tetrasphaera* (*p* = 0.0202) in the old group were lower than those in the young group in 2023 (Figure 4c).

#### 3.2.3. Alpha and Beta Diversities

Based on the alpha diversity analysis, the Simpson index in the 2023 sample group was higher than that in the 2020 sample group, and the Ace index, Chao index, and Shannon index in the 2023 sample group were lower than those in the 2020 sample group, but all the differences did not reach the significance level (Figure S2).

Based on the beta diversity analysis, the present study found that: The contribution rates of PC1 and PC2 were 53.34% and 27.38%, respectively, under weighted Unifrac distance, while the contribution rates of PC1 and PC2 were 37.03% and 12.9%, respectively, under unweighted Unifrac distance. There was a significant inter-group difference (weighted Unifrac: *p* = 0.0060; unweighted Unifrac: *p* = 0.0020) (Figure S3).

### 3.3. Correlation Between Stereotypic Behavior and Gut Microbiota

#### 3.3.1. Correlates of Dominant Phyla

For stereotypic behavior in 2020, among the dominant phyla, (1) a positive correlation was found between the occurrence frequency of stereotypic behavior and the abundances of Proteobacteria (*r* = 0.300, *p* = 0.624) and Fusobacteriota (*r* = 0.100, *p* = 0.837), without reaching the significance level; (2) a negative correlation was found between the occurrence frequency of stereotypic behavior and the abundance of Firmicutes (*r* = −0.300, *p* = 0.624), without reaching the significance level.

**Figure 5:**
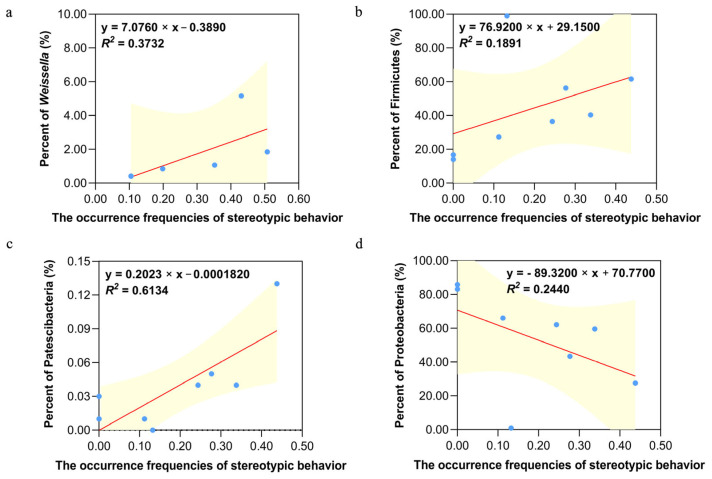
Significant correlation between the occurrence frequencies of stereotypic behavior and abundance of dominant flora taxa. (**a**) Significant correlation between the occurrence frequencies of stereotypic behavior and the abundance of dominant flora taxa (*Weissella*) within the 2020 sample group. (**b**–**d**) Significant correlation between the occurrence frequencies of stereotypic behavior and the abundance of dominant flora taxa ((**b**): Firmicutes, (**c**): Patescibacteria, and (**d**): Proteobacteria) within the 2023 sample group. The vertical axis represents the abundance of dominant flora taxa in the 2020 sample group and 2023 sample group. The horizontal axis indicates the frequencies of stereotypic behavior, which was calculated as a proportion of observations (the number of scans per behavior/total number of scans). Each data point represents one sample. The red line represents the fitted curve and the yellow area is the 95% confidence band.

For stereotypic behavior in 2023, among the dominant phyla, we found that: (1) a positive correlation was found between the occurrence frequency of stereotypic behavior and the abundance of Fusobacteriota (*r* = 0.332, *p* = 0.422), without reaching the significance level; (2) there was a significant positive correlation between the occurrence frequency of stereotypic behavior and the abundance of Firmicutes (*r* = 0.731, *p* = 0.040) (Figure 5b); (3) there was a significant negative correlation between the occurrence frequency of stereotypic behavior and the abundance of Proteobacteria (*r* = −0.731, *p* = 0.040) (Figure 5d).

#### 3.3.2. Correlates of Dominant Genera

For stereotypic behavior in 2020, among the dominant genera, (1) a positive correlation was found between the occurrence frequency of stereotypic behavior and the abundance of *Ligilactobacillus* (*r* = 0.100, *p* = 0.837) and *Clostridium_sensu_stricto_1* (*r* = 0.600, *p* = 0.285), without reaching the significance level; (2) a negative correlation was found between the occurrence frequency of stereotypic behavior and the abundance of *Streptococcus* (*r* = −0.500, *p* = 0.391), *Escherichia-Shigella* (*r* = −0.100, *p* = 0.873), *Klebsiella* (*r* = −0.300, *p* = 0.624), and *Enterococcus* (*r* = −0.600, *p* = 0.285), without reaching the significance level; (3) no linear correlation was found between the occurrence frequency of stereotypic behavior and the abundance of *Cetobacterium* (*r* = 0.000, *p* = 1.000); (4) there was a significant positive correlation between the occurrence frequency of stereotypic behavior and the abundance of *Weissella* (*r* = 0.900, *p* = 0.037) (Figure 5a).

For stereotypic behavior in 2023, among the dominant genera, (1) a positive correlation was found between the occurrence frequency of stereotypic behavior and the abundance of *Escherichia-Shigella* (*r* = 0.323, *p* = 0.435), *Sarcina* (*r* = 0.443, *p* = 0.272), *Streptococcus* (*r* = 0.667, *p* = 0.071), *Cetobacterium* (*r* = 0.204, *p* = 0.628), and *Weissella* (*r* = 0.282, *p* = 0.498), without reaching the significance level; (2) a negative correlation was found between the occurrence frequency of stereotypic behavior and the abundance of *Psychobacteriota* (*r* = 0.667, *p* = 0.071), *Romboutsia* (*r* = −0.024, *p* = 0.955), *Clostridium_sensu_stricto_1* (*r* = −0.524, *p* = 0.183), *Terrisporobacter* (*r* = −0.262, *p* = 0.531), and *Turicibacter* (*r* = −0.257, *p* = 0.509), without reaching the significance level.

#### 3.3.3. Correlates of Non-Dominant Phyla and Genera

For the data of stereotypic behavior in 2020, no significant positive or negative correlation was found between the occurrence frequency on stereotypic behavior and the abundance of any non-dominant phyla and non-dominant genera in 2020 (all *p* > 0.05).

For the data of stereotypic behavior in 2023, there was a significant positive correlation between the occurrence frequency of stereotypic behavior and the abundance of Patescibacteria (*r* = 0.785, *p* = 0.029) (Figure 5c) and no significant correlation between the occurrence frequency of stereotypic behavior and the abundance of other non-dominant phyla in 2023 (all *p* > 0.05).

For the stereotypic behavior in 2023, no significant correlation was found between the abundance of any non-dominant genera and the occurrence frequency of stereotypic behavior in 2023 (all *p* > 0.05).

## 4. Discussion

Our study found that the stereotypic behavior of captive sun bears included pacing behavior, and pacing behavior is the main manifestation of stereotypic behavior in captive sun bears. This finding is consistent with previous related results for the same species [18], other Ursidae species (e.g., polar bear (*Ursus maritimus*) [22], and non-Ursidae ursid species (e.g., tiger (*Panthera tigris*) [38]). Using pacing behavior as an example, we aim to reveal the potential association between animal behavior and gut microbiota.

Seasonal differences have been found in the occurrence frequency of stereotypic behavior among captive animals (e.g., giant panda (*Ailuropoda melanoleuca*) [5,39]). The present study found significant seasonal differences in stereotypic behavior in captive sun bears; the occurrence frequency of stereotypic behavior in spring was higher than that in both summer and autumn, which aligns with previous results for the same species [37] and other Ursidae species (e.g., polar bear (*Ursus maritimus*) [40]). Meanwhile, some studies reported that captive giant pandas living apart showed an increase in stereotypic behavior, which was mainly due to the inability to have sex when living separately [5,39]. Similarly, the environment of solitary rearing in this study was not conducive to reproductive communication in spring, which may have also caused the increase in the occurrence frequency of stereotypic behavior.

In this study, the average occurrence frequency of resting behavior in summer was higher than that in spring and autumn, which is consistent with previous studies on the same species [32], other Ursidae species (e.g., polar bear (*Ursus maritimus*) [40] and giant panda (*Ailuropoda melanoleuca*) [39,41]), and non-Ursidae species (e.g., lion (*Panthera leo*) [42]). Meanwhile, this study found that the average occurrence frequency of stereotypic behavior in summer was lower than that in spring and autumn. It is well known that the temperature in summer is relatively higher compared to those of other seasons. It has been found that the active behavior decreased with the increase in temperature [40]. It is likely that the decrease in stereotypic behavior and the increase in resting behavior of sun bears in summer belongs to their adaptation to a hot climate.

For the inter-year behavioral comparison, it was found that the occurrence frequency of both resting behavior and stereotypic behavior in autumn 2020 were higher than that in autumn 2023, while the occurrence frequency of active behavior presented the opposite effect. Kelly et al. (2015) found that polar bears have a higher activity tendency when the temperature is relatively low [40], and Gandia et al. (2023) found that the activity of giant pandas decreases with the increase in the minimum temperature [43]. In autumn 2023, the observation time was from November 18 to November 30, with an average temperature of 4 °C. The observation time in autumn 2020 was from October 5 to November 11, with an average temperature of 13 °C. Therefore, sun bears in this study showed a higher active behavior in the relatively cold autumn of 2023, which may be due to behavioral strategies in order to reduce the cost of physiological thermoregulation to better adapt to low temperatures [44]. In addition, in this study, a row of poplar trees was planted in front of the cage where sun bears live. During the autumn observation period in 2020, the number of poplar tree leaves that dropped decreased, while in the cooler autumn of 2023, a large number of poplar tree leaves fell. The sun bears also exhibited feeding behavior on fallen poplar leaves. Poplar leaf litter is an unconventional potential feed resource [45,46,47]. Dunham et al. (2023) found that feeding natural fallen leaves can help captive animals increase their foraging time and reduce stereotypic behavior, thus promoting positive welfare [48]. The relatively higher supply of poplar leaf resources in 2023 could have helped to increase the related feeding time and active behavior, thereby reducing the occurrence of stereotypic behavior to a certain extent, resulting in a lower frequency of stereotypic behavior in autumn 2023 compared to autumn 2020.

With regard to the gut microbiota, our study found that the common dominant bacteria of 2020 and 2023 sample groups included Firmicutes, Proteobacteria, and Fusobacteriota, which is consistent with previous results for the same species [32], other Ursidae species (e.g., Andean bear (*Tremarctos ornatus*) [49]), and non-Ursidae species (e.g., Yunnan snub-nosed monkey (*Rhinopithecus bieti*) [50], platypus (*Ornithorhynchus anatinus*) [51], and the Père David’s deer (*Elaphurus davidianus*) [52]). The main functions of Firmicutes contribute to the metabolism of the plant-based diet and play a role in energy intake and weight gain in large carnivores [53,54]. Proteobacteria is found to be related to the degradation of cellulose and can help hosts utilize carbon sources and play an important role in energy accumulation [26,55]. Fusobacteriota can better assist the body in digesting fat and participate in the homeostasis maintenance and metabolic function of intestinal flora [56,57]. The diet of captive sun bears in this study included both meat and plants, and therefore, these common dominant bacteria (Firmicutes, Proteobacteria, and Fusobacteriota) help them to digest protein and cellulose effectively.

The common dominant bacterial genera of the 2020 and 2023 sample groups in this study included *Escherichia-Shigella*, *Streptococcus*, *Clostridium_sensu_stricto_1*, *Weissella*, and *Cetobacterium*, which partially align with previous results for the same species [58]. It has been found that *Escherichia-Shigella*, *Clostridium_sensu_stricto_1*, *Enterococcus*, and *Turicibacter* are associated with the degradation of cellulose [58,59,60]. The findings for the sun bears in the studies by Li (2014) and Yuan et al. (2019) on dominant bacterial genera do not contain the genus *Weissella* while containing *Enterococcus* and *Turicibacter* [58,61]. Due to the fact that the diet of sun bears in the study by Yuan et al. (2019) does not include meat, such as chicken racks, but instead includes vegetables, such as cucumbers and potatoes [58], it is speculated that inconsistent findings on dominant genera may be due to different diets. It was found that *Cetobacterium* participate in host vitamin metabolism, produce vitamin B12 and antimicrobial peptides, and also have the function of fermenting peptide carbohydrates [62], and the abundance of *Cetobacterium* helps to digest carbohydrates such as wotou. The genus *Weissella* can ferment sugars and produce lactic acid [63]; it also produces β- Glucosidase to promote cellulose degradation [64]. Therefore, as the dominant genus, *Weissella* could help the host to digest carbohydrates, such as steamed buns, fruits, and vegetables.

Age is one of the main factors affecting animal gut microbiota, and the abundance of some bacterial genera may change with age [58,65]. Compared to the 2020 and 2023 sample groups, the abundances of *Stenotrophomonas*, *unclassified_f__Sphingomonadaceae*, *unclassified_f__Enterobacteriaceae, Ligilactobacillus*, *Klebsiella*, *Citrobacte*, *unclassified_d__Bacteria*, and *Pseudomonas* in the 2023 sample group were lower than those in the 2020 sample group; and the abundance of *Sarcina* in the 2023 sample group was significantly higher than that in the 2020 sample group. Pedro et al. (2024) found that the abundance of the genus *Ligilactobacillus* decreased in mouse gut microbiota with host aging [66]. Wang (2020) found that increasing age leads to a decrease in the ability to metabolize carbohydrates [67], while the genera *Klebsiella* and *Citrobacter* have the ability to break down lactose [65]. Wang (2020) and Yang (2023) found that, for captive giant pandas, the abundance of the genus *Sarcina* increased with age, while the abundances of and Enterobacteriaceae decreased with age [67,68]. Our study found the same changes for those bacterial genera mentioned above when making a comparison of two sample groups in different years. Compared to the old and young groups, we found *Actinomyces* was enriched in the old group. *Actinomyces* has been reported previously to be cellulose decomposers [69]; so, the increase of *Actinomyces* may be due to the observation that the old group preferred to feed on poplar leaves compared to the younger group. Therefore, it is speculated that these changes have a potential relationship with inter-group age differences in this study.

In this study, the occurrence frequency of stereotypic behavior in captive sun bears exhibited significant positive correlations with both Firmicutes and Patescibacteria, and a significant negative correlation with Proteobacteria. Recent research has demonstrated an enrichment in the phylum Firmicutes in patients with autism spectrum disorder (ASD) [70,71], while the phylum Patescibacteria has been found to be enriched in rats with ASD-like symptoms [72]. Kang et al. (2017) found that the amino acid metabolism pathways in ASD patients generally increase compared to the control groups [73]. Notably, Firmicutes are positively correlated with amino acid metabolism, whereas Proteobacteria show a negative correlation [74,75]. The relevant findings in this study are consistent with those of previously published studies on the relationship between corresponding phyla and ASD in humans and rats.

Through a comprehensive comparison of the correlation characteristics between stereotypic behavior and gut microbiota across the two sample groups, our study revealed that the correlation between Fusobacteriota, *Weissella*, and stereotypic behavior remained consistent across sampling years. This observation leads to the preliminary speculation that these phyla and genera may have a potential association with the stable expression of stereotypic behavior. Furthermore, a significant positive correlation was observed between the occurrence frequency of stereotypic behavior and the abundance of *Weissella*. Previous studies have indicated that *Weissella* plays a role in endogenous ethanol production [76]. Ethanol may damage the intestinal barrier and alter its permeability, leading to metabolic endotoxemia. Fung et al. (2017) found that abnormal intestinal permeability may lead to increased levels of lipopolysaccharides (LPSs) in the blood [77]. Fusobacteriae are notable, amongst Gram-negative anaerobic bacteria, for their ability to invade the host as primary pathogens, and LPSs are one of the determinants of their considerable virulence [78]. LPSs induced cognitive impairment and neuroinflammation via microglia activation by activating the NF-kB signaling pathway [79]. This result may lead to stereotypic behavior happening. Numerous studies have demonstrated that interventions targeting gut health and mitigating inflammation may effectively alleviate stereotypic behaviors in captive animals. However, the current research on sun bears presents certain limitations. Further comprehensive investigations are essential to fully elucidate the underlying mechanisms and develop robust, effective treatment strategies.

## 5. Conclusions

Overall, the analysis of captive sun bears in zoos conducted in this study provides the first evidence of a potential association between gut microbiota and stereotypic behavior in captive endangered species. The findings reveal seasonal and inter-annual variations in the stereotypic behavior of captive sun bears, with a certain correlation between the frequency of stereotypic behavior and gut microbiota. Additionally, the key bacterial genera were ide. However, it is important to acknowledge that the current research on related phyla and genera does not sufficiently explain the observed correlation between stereotypic behavior and specific phyla or genera. Therefore, the correlation and mechanism need further verification and exploration in future studies. It is recommended to further increase the sample size and conduct long-term observations of stereotypic behavior across multiple sites to comprehensively compare the correlation characteristics between stereotypic behavior and gut microbiota under different spatiotemporal environments, to provide comprehensive scientific references for the ex situ protection of and welfare improvement in these endangered species.

## Figures and Tables

**Table 1:** Focal individuals and observation data.

Individuals Involved in Data Collection			Days of Observation/The Total Number of Scans				
Name	Sex	Age in Autumn 2020	Autumn 2020 (October 5 to November 11)	Spring 2023 (April 15 to May 28)	Summer 2023 (June 3 to July 2)	Autumn 2023 (November 18 to November 30)	Total
TT	Female	1 (Young)	0/0	0/0	0/0	5/294	5/294
GG	Female	3 (Young)	0/0	0/0	0/0	5/294	5/294
LL	Female	8 (Young)	10/582	7/536	4/227	5/294	26/1639
BB	Male	9 (Young)	10/582	7/536	4/227	5/294	26/1639
ZZ	Male	19 (Old)	10/582	7/536	4/227	5/294	26/1639
SJZ	Female	>12 (Old)	10/582	7/536	4/227	5/294	26/1639
DL	Female	>12 (Old)	0/0	6/475	4/227	5/294	15/996
HH	Female	26 (Old)	10/582	7/536	4/227	5/294	26/1639

**Table 2:** Classification and definition of behaviors.

Category	Behavior	Definition
Stereotypic behavior	Pacing	Back and forth, or perimeter traveling in a repetitive, sustained, stereotypic pattern. Must travel the same route at least 3 times.
Rest behavior	Rest, drowsy/asleep	Sitting or lying with body motionless for at least 30 s and/or eyes closed; does not appear alert.
	Rest alert	Sitting or lying with eyes open; appears alert.
Active behavior	Locomotion	Walk, run, climb, change stance, stretch, sniff, dig, scratch, and rub.
	Feeding and drinking	Ingestion of edible material and consumption of water.
	Conspecific interaction	Threaten, chase, attacking, play fighting, and copulate.
	Excretion	The process of removing waste and urine from the body through the anus or urethra.
Other	Obscure	Any behavior not falling into one of the above categories.

Note: This table refers to Refs. [12,33,34].

## Data Availability

DNA sequencing data were deposited in the NCBI Sequence Read Archive (SRA) database under PRJNA1110582.

## Supplementary Materials

The following supporting information can be downloaded at: https://www.mdpi.com/article/10.3390/ani15030435/s1. Figure S1: Venn diagram of the two sample groups at the phylum (a), genus (b), and OTU (c) levels; Figure S2: Alpha diversity analysis of the two sample groups (a. Ace index; b. Chao index; c. Shannon index; d. Simpson index); Figure S3: Beta diversity analysis of the two sample groups (a. Weighted UniFrac analysis; b. Unweighted UniFrac analysis); Table S1: Adult sun bear diet composition; Table S2: The information of all fecal samples; Table S3: Data on the dominant phyla and genera for the two sample groups.
