# Supplementary material for: Linking Gut Microbiota and Stereotypic Behavior of Endangered Species Under Ex Situ Conservation: First Evidence from Sun Bears
Source: Animals (Basel). 2025 Feb 4;15(3):435. doi: 10.3390/ani15030435 (PMC11815909; doi:10.3390/ani15030435)

1 Supplementary Tables

Table S1. Aduit sun bears diet composition

**Food types**	Male	Female
Steamed buns	2 kg	1.5 kg
Chicken racks	1 kg	0.5 kg
Vegetable (carrots, sweet potatoes, pumpkins, cucumbers, tomatoes)	1.5-2 kg	1-1.5 kg
Eggs	4-5 piece	3-4 piece
Seasonal fruit (apples, bananas, peaches, grapes, watermelons)	1-1.5 kg	0.5-1 kg

Table S2. The information of the all fecal sample

Sample number	Sample source	Collection time
GG-1-F-Y	GG	September 24, 2020
LL-1-F-Y	LL	September 24, 2020
BB-1-M-Y	BB	September 24, 2020
ZZ-1-M-O	ZZ	September 24, 2020
SJZ-1-F-O	SJZ	September 24, 2020
HH-1-F-O	HH	September 24, 2020
TT-2-F-Y	TT	November 25, 2023
GG-2-F-Y	GG	November 25, 2023
LL-2-F-Y	LL	November 25, 2023
BB-2-M-Y	BB	November 25, 2023
ZZ-2-M-O	ZZ	November 25, 2023
SJZ-2-F-O	SJZ	November 25, 2023
DL-2-F-O	DL	November 25, 2023
HH-2-F-O	HH	November 25, 2023

Table S3. Data on dominant phyla and genera for two sample groups

Group name	Dominant phyla		Dominant genera	
	**Phylum name**	**Percentages**	**Genus name**	**Percentages**
2020 sample group	Firmicutes	63.39%	*Streptococcus*	46.84%
	Proteobacteria	35.333%	*Escherichia-Shigella*	30.65%
	Fusobacterium	1.06%	*Ligilactobacillus*	5.91%
			Clostridium_sensu_stricto_1	3.93%
			Enterococcus	3.43%
			Klebsiella	3.29%
			Weissella	1.56%
			Cetobacterium	1.06%
2023 sample group	Firmicutes	53.58%	*Escherichia-Shigella*	44.73%
	Proteobacteria	43.95%	*Sarcina*	19.09%
	Fusobacterium	2.08%	*Streptococcus*	13.44%
			Psychrobacter	8.20%
			Romboutsia	3.01%
			Clostridium_sensu_stricto_1	2.55%
			Turicibacter	1.60%
			Cetobacterium	1.57%
			Terrisporobacter	1.34%
			Weissella	1.25%

2 Supplementary Figures

Figure S1. Venn diagram of two sample groups at phylum (a), genus (b) and OTU (c) levels

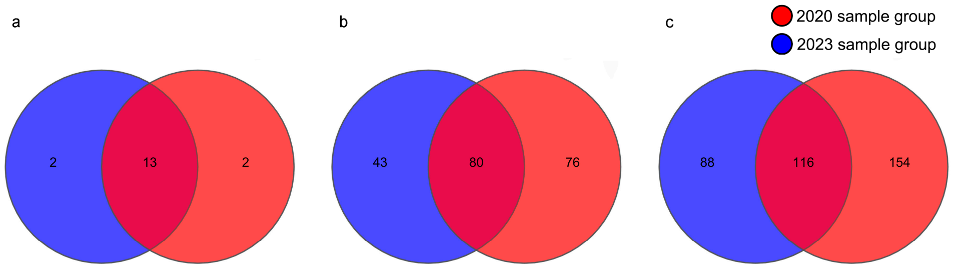

Figure S2. Alpha diversity analysis of two sample groups (a. Ace index; b. Chao index; c. Shannon index; d. Simpson index)

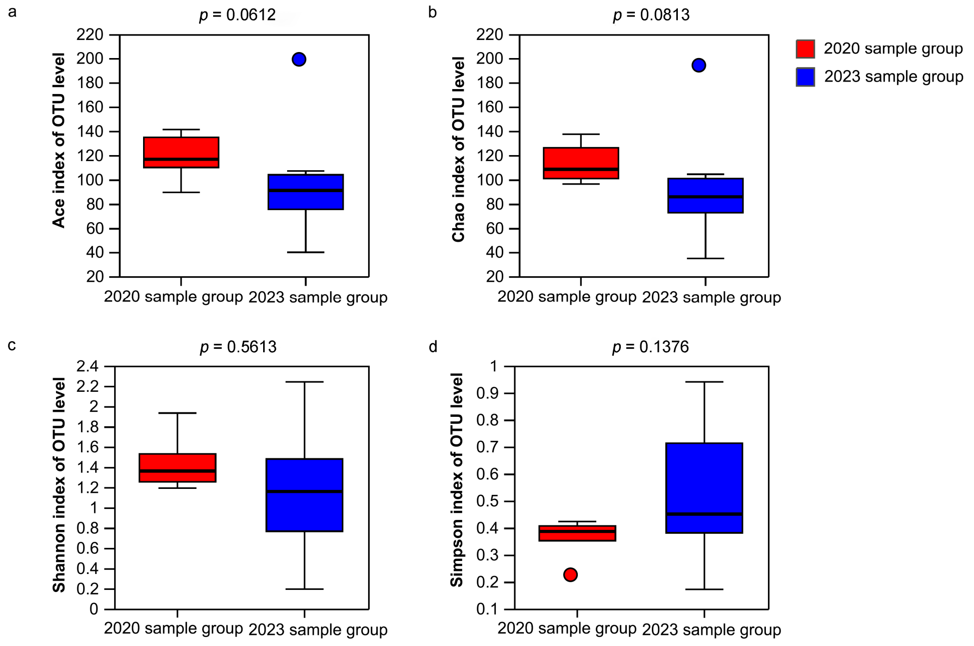

Figure S3. Beta diversity analysis of two sample groups (a. Weighted UniFrac analysis; b. Unweighted UniFrac analysis)

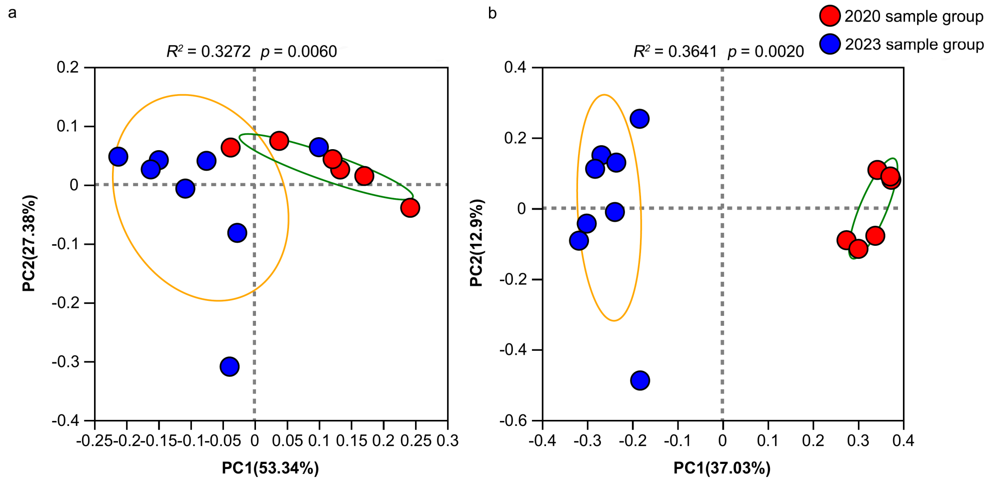

Supplement: Supplementary file 1 [file animals-15-00435-s001.zip › animals-3303231-supplementary.pdf]
